# Rhino-Orbital Mucormycosis Presenting With Central Retinal Artery Occlusion: A Case Report

**DOI:** 10.7759/cureus.88020

**Published:** 2025-07-15

**Authors:** Irem Sena Sarac, Yasemin Aksu, Dilan Yildiz, Ahu Yilmaz

**Affiliations:** 1 Ophthalmology, Prof. Dr. Cemil Taşçıoğlu City Hospital, Istanbul, TUR

**Keywords:** amphotericin b, central retinal artery occlusion, diabetes mellitus, mucormycosis, rhino-orbital mucormycosis

## Abstract

Rhino-orbital-cerebral mucormycosis (ROCM) is a severe fungal infection caused by opportunistic fungi of the order Mucorales, most commonly Rhizopus oryzae. It typically occurs in patients with diabetes mellitus or those who are immunocompromised. The diagnosis is based on histopathological, microbiological, clinical, and radiological findings. Early diagnosis is crucial, and management includes antifungal therapy and surgical debridement. In this case report, we report a 64-year-old diabetic female patient who presented with rhino-orbital mucormycosis. The patient had central retinal artery occlusion in the left eye, left cranial nerve palsies, and ethmoidal and maxillary sinusitis. The fungal culture examination resulted in Rhizopus oryzae. The patient received antifungal treatment and underwent left orbital exenteration and endoscopic sinus surgery. Despite antifungal treatment and early extensive surgical debridement, the patient died during the second month of intensive care.

## Introduction

Mucormycosis is a life-threatening opportunistic fungal infection [1]. It can be acquired through inhalation of fungal spores or direct inoculation following skin disruption, such as burns or trauma [1,2]. Major risk factors include uncontrolled diabetes mellitus, hematological malignancies, and immunosuppressive therapy [2,3]. Mucormycosis is an angio-invasive fungal infection, frequently causing vascular thrombosis and tissue necrosis [3]. The most common clinical presentations of mucormycosis are rhino-orbital-cerebral mucormycosis (ROCM) and pulmonary mucormycosis [4]. We present the case of a 64-year-old diabetic female patient diagnosed with rhino-orbital mucormycosis, based on physical examination, orbital and cerebral imaging, clinical correlation, and tissue culture. She underwent orbital exenteration as part of her management. 

## Case presentation

A 64-year-old female patient presented to the emergency department complaining of dizziness, headache, and decreased vision in the left eye for the past week. She was subsequently referred to our department for further evaluation. 

**Figure 1:**
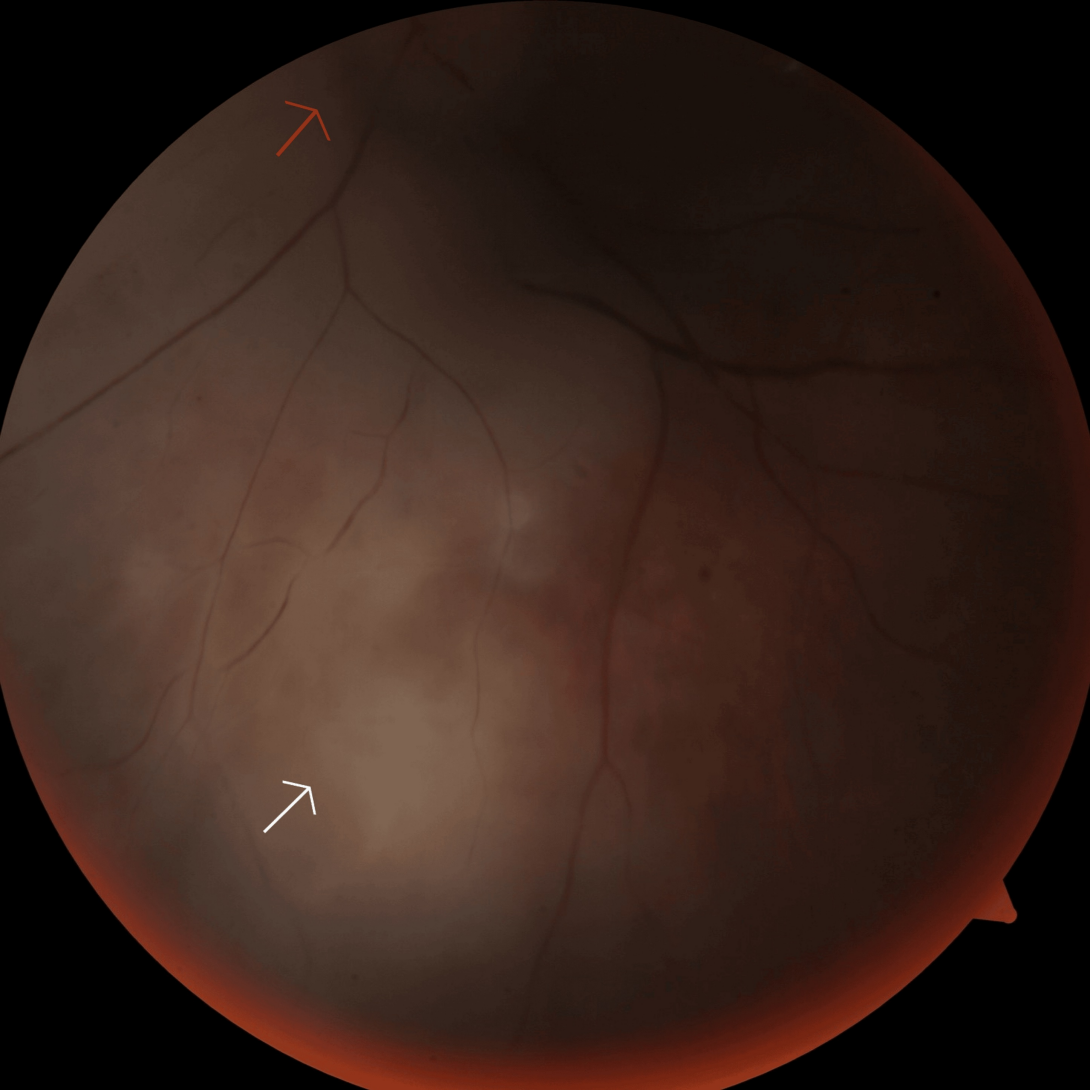
Fundus photo showing ischemic retinal areas (white arrow) and a pale optic disc (red arrow).

On initial examination, the best-corrected visual acuity in the right eye was 6/60, and the left eye had no light perception. The left pupil was non-reactive to light. The right eye’s anterior segment examination was normal. Fundus examination of the right eye revealed retinal hemorrhages and exudates consistent with diabetic retinopathy, and the optic disc appeared normal. There was ptosis, mild proptosis, and chemosis in the left eye. No eyelid edema or hyperemia was noted at that time. The left eye's extraocular motility was limited in all directions. The anterior segment examination of the left eye was normal. Fundus examination of the left eye revealed an edematous optic disc, retinal hemorrhages and exudates consistent with diabetic retinopathy, and retinal pallor with a cherry-red spot at the fovea consistent with central retinal artery occlusion (Figure 1). On the second day of hospitalization, eyelid edema and hyperemia developed in the left eye. 

**Figure 2:**
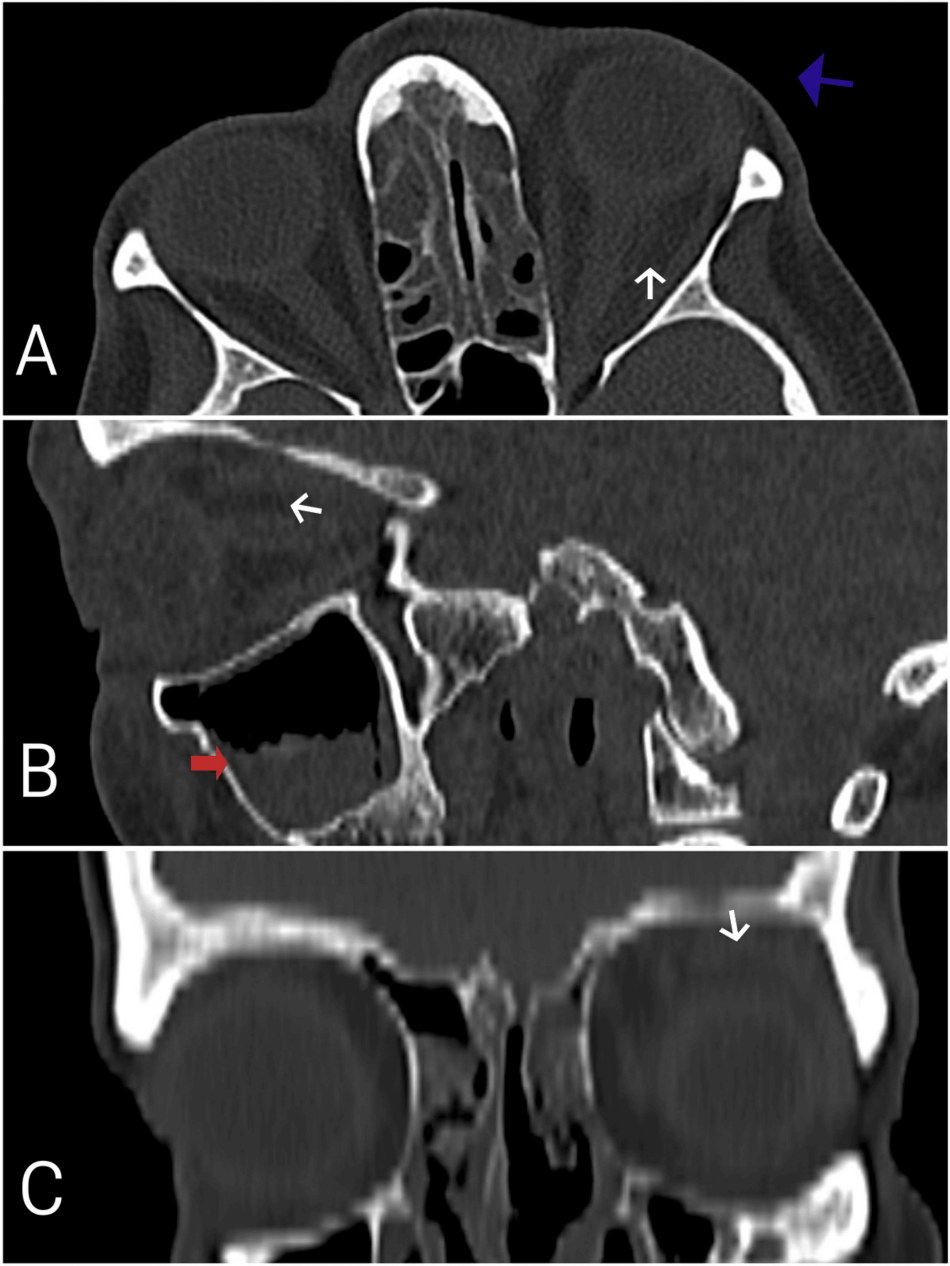
Axial (A), sagittal (B), and coronal (C) sections of a bilateral non-contrast orbital CT scan at the patient's initial hospital admission showing left orbital cellulitis (white arrows), proptosis of the left eye (blue arrow), and marked ethmoidal and maxillary sinusitis (red arrow).

**Figure 3:**
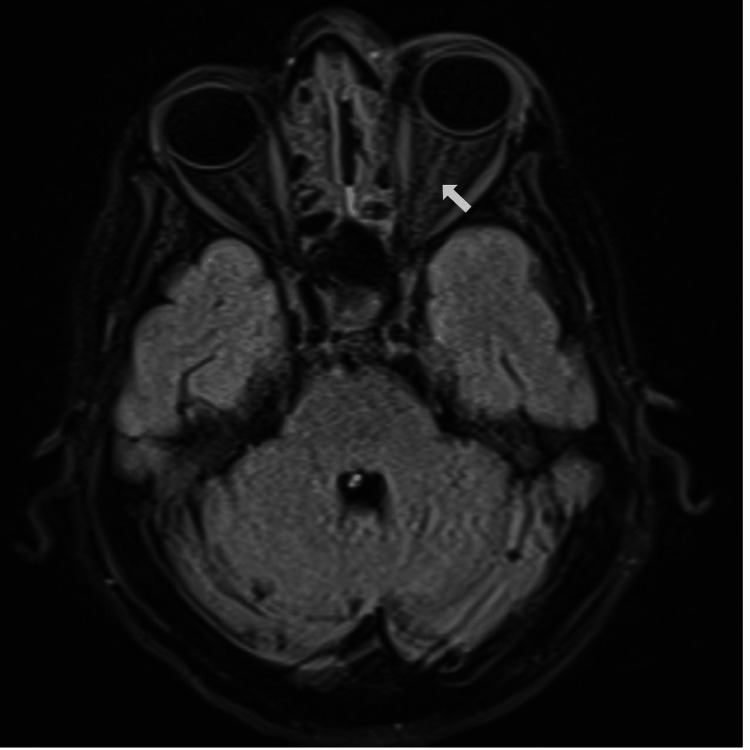
Axial section of a bilateral non-contrast orbital MRI at the patient's initial hospital admission showing increased inflammatory T2 signal around the left optic nerve (white arrow).

Laboratory tests revealed leukocytosis with neutrophil predominance, elevated C-reactive protein (CRP), hyperglycemia, and elevated hemoglobin A1c (HbA1c) (Table 1). Non-contrast orbital and paranasal sinus CT scan revealed left orbital cellulitis, proptosis of the left eye, and severe ethmoidal and maxillary sinusitis (Figure 2). Histopathological examination of the nasal mucosa biopsy revealed broad, non-septate fungal hyphae, and culture results identified Rhizopus oryzae. No black necrotic tissue was observed in the nasal and sinus mucosa and skin. Blood cultures showed no growth. Cranial MRI demonstrated maxillary and ethmoidal sinusitis, as well as increased T2 signal intensity surrounding the left optic nerve, consistent with inflammation (Figure 3). 

**Table 1:** Laboratory findings at admission. CRP: C-reactive protein; WBC: white blood cells

Parameters	Admission	Normal range
CRP (mg/L)	215	<5
Serum glucose (mg/dL)	321	74-100
Hemoglobin A1c (%)	13.5	<6.5
WBC (x10^3^/µL)	19.6	3.8-10
Neutrophils (x10^3^/µL)	16.24	1.56-6.13

**Figure 4:**
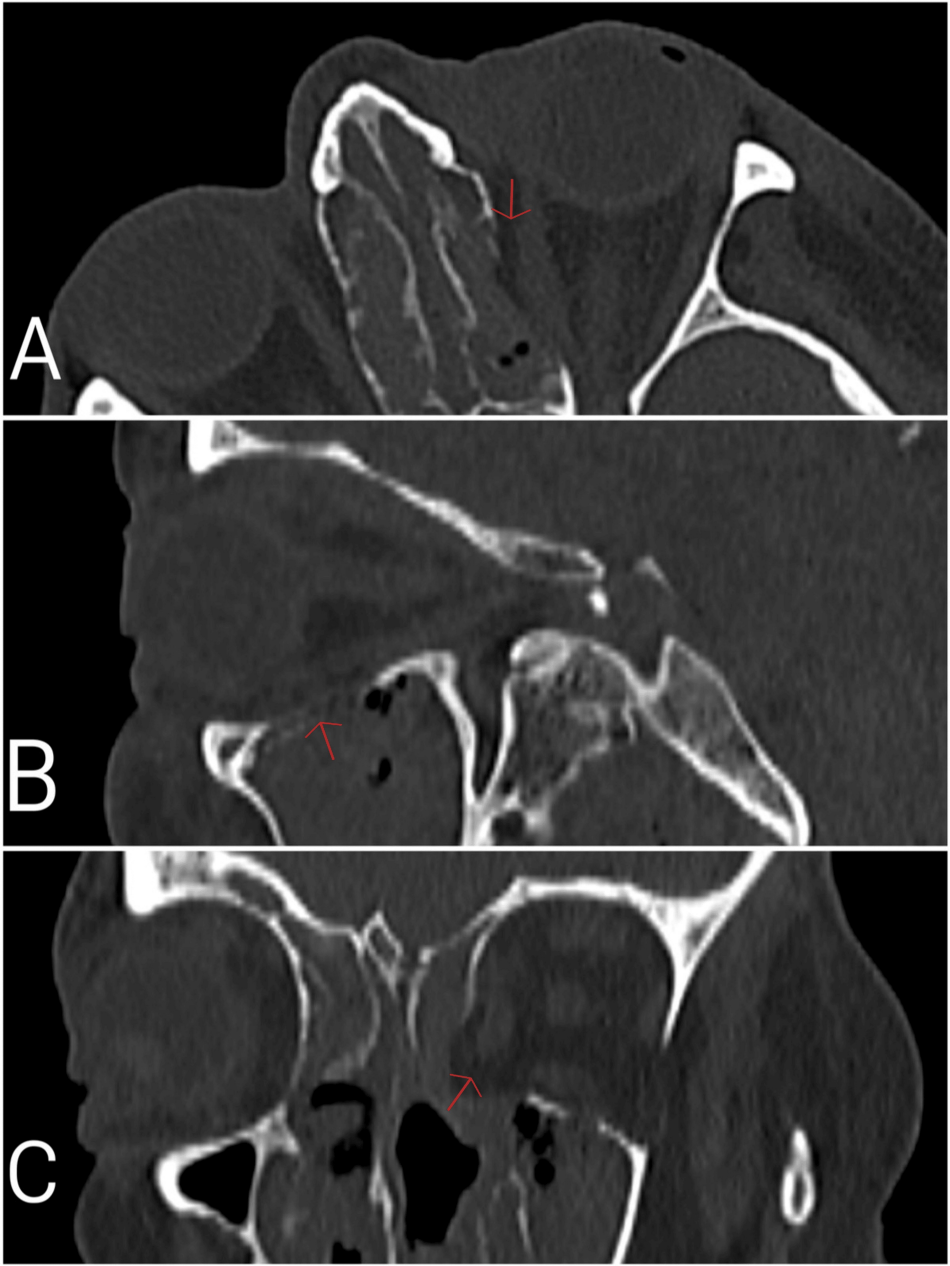
Axial (A), sagittal (B), and coronal (C) sections of a bilateral non-contrast orbital CT scan on the fourth day of the patient’s hospital admission showing bone defects in the medial and inferior walls of the left orbit (red arrows).

On the first day of admission, liposomal amphotericin B was initiated at a dose of 350 mg (5 mg/kg/day) following consultation with the infectious diseases team. On the second day, endoscopic sinus surgery was performed. On the fourth day, an orbital CT scan showed a bone defect in the left orbital medial wall (Figure 4). Left orbital exenteration was performed on the fifth day. The patient was subsequently monitored in the intensive care unit postoperatively. On the 10th day, the dose of amphotericin B was increased to 700 mg (10 mg/kg/day). One week after the exenteration, CRP decreased to 50 mg/L. However, two weeks after the initial sinus surgery, a follow-up CT scan showed progression of sinusitis, and a second endoscopic sinus surgery was performed. During follow-up, no signs of infection or inflammation were observed in the right eye or orbit. Postoperative control CT scan following the second sinus surgery confirmed disease stabilization. Despite aggressive treatment, blood cultures later grew pan-resistant Klebsiella and Pseudomonas aeruginosa, and the patient developed acute renal failure. The patient died on the 64th day of hospitalization. 

## Discussion

ROCM is the most common in diabetic patients, especially those with poor glycemic control [5]. Proptosis, ophthalmoplegia, and ptosis are frequently observed in patients with ROCM [5]. In our case, the patient presented with ptosis, unilateral facial pain and edema, and cranial nerve palsies. The presence of ophthalmoplegia, headache, ptosis, and proptosis suggested cavernous sinus thrombosis as a differential diagnosis. No cavernous sinus thrombosis was observed in CT and CT angiography. MR venography was performed, which showed no dilation of the superior ophthalmic veins, thereby excluding cavernous sinus thrombosis. 

Mucormycosis is an angiotropic fungal infection that invades the internal elastic lamina of blood vessels, leading to angioinvasion, hemorrhagic necrosis, and vascular thrombosis, particularly affecting arteries, veins, and lymphatics, through both mechanical compression and toxin-mediated damage [6]. Vision loss may be due to central retinal artery occlusion or orbital apex involvement affecting the optic nerve function [6]. In our case, central retinal artery occlusion was suspected to have occurred either due to compression of the ophthalmic artery from soft tissue invasion of the orbital apex or as a direct result of angioinvasion by the fungus. 

The diagnosis of mucormycosis is based on direct microscopy, fungal culture, and histopathology of clinical specimens [7]. A requirement for identifying mucormycosis as a confirmed infection is the presence of broad, non-septate, or pauci-septate hyphae with wide-angle branching in affected tissue and evidence of tissue invasion [7]. In our case, non-septate fungal hyphae were observed in the nasal mucosal biopsy, and the culture grew Rhizopus oryzae complex. 

First-line treatment with liposomal amphotericin B at a dose of 5-10 mg/kg/day for a minimum of six to eight weeks is highly recommended for all patterns of organ involvement [8]. If significant renal toxicity develops, dose reduction may be considered; however, doses below 5 mg/kg/day are only weakly recommended [8]. The two major complications associated with intravenous amphotericin B therapy are hypokalemia and acute kidney injury [7]. In our case, intravenous liposomal amphotericin B at 5 mg/kg/day was initiated on the first day of admission, and surgery was performed within four days. In addition, acute renal failure developed during the follow-up period of our patient. 

Early and extensive surgical debridement is recommended in ROCM to reduce fungal burden and should be repeated if necessary to achieve local control and reduce mortality [7]. Despite timely antifungal therapy and aggressive surgical management, our patient died during the second month of intensive care. 

## Conclusions

Mucormycosis infection should be considered as a differential diagnosis in diabetic patients presenting with sudden vision loss and cranial nerve palsies. Mucormycosis is a rapidly progressing, devastating infection that requires rapid diagnosis and management by a multidisciplinary team. 
